# Genomic regions associated with susceptibility to Barrett’s esophagus and esophageal adenocarcinoma in African Americans: The cross BETRNet admixture study

**DOI:** 10.1371/journal.pone.0184962

**Published:** 2017-10-26

**Authors:** Xiangqing Sun, Apoorva K. Chandar, Marcia I. Canto, Prashanthi N. Thota, Malcom Brock, Nicholas J. Shaheen, David G. Beer, Jean S. Wang, Gary W. Falk, Prasad G. Iyer, Julian A. Abrams, Medha Venkat-Ramani, Martina Veigl, Alexander Miron, Joseph Willis, Deepa T. Patil, Ilke Nalbantoglu, Kishore Guda, Sanford D. Markowitz, Xiaofeng Zhu, Robert Elston, Amitabh Chak

**Affiliations:** 1 Department of Population and Quantitative Health Sciences, Case Western Reserve University, Cleveland, OH, United States of America; 2 Division of Gastroenterology and Hepatology, University Hospitals Cleveland Medical Center, Case Western Reserve University School of Medicine, Cleveland, OH, United States of America; 3 Division of Gastroenterology and Hepatology, Johns Hopkins Medical Institutions, Baltimore, MD, United States of America; 4 Department of Gastroenterology and Hepatology, Cleveland Clinic, Cleveland, OH, United States of America; 5 Department of Cardiology and Thoracic Surgery, Johns Hopkins Medical Institutions, Baltimore, MD, United States of America; 6 Center for Esophageal Diseases & Swallowing, University of North Carolina at Chapel Hill School of Medicine, Chapel Hill, NC, United States of America; 7 Thoracic Surgery, Department of Surgery, University of Michigan, Ann Arbor, MI, United States of America; 8 Division of Gastroenterology, Washington University School of Medicine, St Louis, MO, United States of America; 9 University of Pennsylvania Perelman School of Medicine, Philadelphia, PA, United states of America; 10 Division of Gastroenterology and Hepatology, Mayo Clinic, Rochester, MN, United States of America; 11 Department of Medicine, Columbia University Medical Center, New York, NY, United States of America; 12 Division of General Medical Sciences (Oncology), Case Comprehensive Cancer Center, Case Western Reserve University School of Medicine, Cleveland, OH, United States of America; 13 Department of Genetics and Genome Sciences, Case Western Reserve University School of Medicine, Cleveland, OH, United States of America; 14 Department of Pathology, University Hospitals Case Medical Center, Case Western Reserve University School of Medicine, Cleveland, OH, United States of America; 15 Department of Pathology, Cleveland Clinic, Cleveland, OH, United States of America; 16 Department of Pathology and Immunology, Washington University School of Medicine, St. Louis, MO, United States of America; 17 Division of Oncology and Case Comprehensive Cancer Center, Case Western Reserve University School of Medicine, Cleveland, OH, United States of America; Shiga University of Medical science, JAPAN

## Abstract

**Background:**

Barrett’s esophagus (BE) and esophageal adenocarcinoma (EAC) are far more prevalent in European Americans than in African Americans. Hypothesizing that this racial disparity in prevalence might represent a genetic susceptibility, we used an admixture mapping approach to interrogate disease association with genomic differences between European and African ancestry.

**Methods:**

Formalin fixed paraffin embedded samples were identified from 54 African Americans with BE or EAC through review of surgical pathology databases at participating Barrett’s Esophagus Translational Research Network (BETRNet) institutions. DNA was extracted from normal tissue, and genotyped on the Illumina OmniQuad SNP chip. Case-only admixture mapping analysis was performed on the data from both all 54 cases and also on a subset of 28 cases with high genotyping quality. Haplotype phases were inferred with Beagle 3.3.2, and local African and European ancestries were inferred with SABER plus. Disease association was tested by estimating and testing excess European ancestry and contrasting it to excess African ancestry.

**Results:**

Both datasets, the 54 cases and the 28 cases, identified two admixture regions. An association of excess European ancestry on chromosome 11p reached a 5% genome-wide significance threshold, corresponding to -log_10_(P) = 4.28. A second peak on chromosome 8q reached -log_10_(P) = 2.73. The converse analysis examining excess African ancestry found no genetic regions with significant excess African ancestry associated with BE and EAC. On average, the regions on chromosomes 8q and 11p showed excess European ancestry of 15% and 20%, respectively.

**Conclusions:**

Chromosomal regions on 11p15 and 8q22-24 are associated with excess European ancestry in African Americans with BE and EAC. Because GWAS have not reported any variants in these two regions, low frequency and/or rare disease associated variants that confer susceptibility to developing BE and EAC may be driving the observed European ancestry association evidence.

## Introduction

Esophageal adenocarcinoma (EAC) is the seventh leading cause of death in U.S. males [1] and one of the deadliest cancers worldwide, with 5-year survival rates lower than 20% [2], and in the United States the incidence rate has increased dramatically up to 7-fold over the past three decades [3,4]. EAC tends to arise from Barrett’s esophagus (BE), which replaces the squamous epithelium with columnar-lined metaplastic epithelium in the lower esophagus during healing reflux esophagitis and may progress to dysplasia [5]. BE and EAC are rare in African Americans (AA), with the majority of cases occurring in European Americans (EA) [6–12]. Although EAC occurs at least five-fold more frequently in EAs than AAs [13], the distribution of known risk factors for BE and EAC (e.g. GERD [14], obesity [15,16], etc) are at least as common in AA as EA, suggesting another basis for the racial differences.

Hypothesizing that this racial/ethnical difference in prevalence might represent a genetic susceptibility, we used an admixture mapping approach to interrogate disease association with genetic differences in European and African ancestry using a multi-institutional sample of AA patients with EAC or BE. Although our sample size is limited because of the rareness of BE in AA patients, we demonstrate here–the first and the only admixture mapping study of BE and EAC we know of–how this approach can efficiently find regions of genetic susceptibility for this disease.

## Materials and methods

### Samples and genotyping

The study was approved by the Institutional Review Board of the University Hospitals of Cleveland. Formalin fixed paraffin embedded (FFPE) samples were identified from 54 AA patients with EAC or BE through review of surgical pathology databases at eight participating BETRNet institutions. The samples were coded and had no identifying information. The codes and identifying information were kept secure at each individual institution and were only available to the institutional investigator who identified the subject, and were not available to any other research personnel. Germline DNA was extracted from normal tissue of the samples, generally from squamous epithelium; in 9 cases the germline DNA was obtained from other non-neoplastic tissues (5 gastric, 2 colonic, 1 vocal cord, and 1 adipose tissue biopsies). DNA samples were genotyped on the Illumina OmniQuad SNP chip. We considered BE and EAC to be part of the same trait, theorizing that at least a proportion of EAC arose from BE.

Of the 54 patient samples, 30 had a genotyping call rate > 0.95 and, after excluding 2 samples because of very low heterozygosity rates (outside the range of 3 standard deviations from the mean heterozygosity), we formed an enriched sample set of 28 samples.

There were 2.3 million SNPs genotyped, but upon additional processing following instructions from Illumina technologists, and restricting SNPs to call rates greater than 96.4%, 378,711 SNPs were available for data analysis.

For the admixture mapping study, the two ancestry populations were selected from the 1000 genome project [17], which included genotype data on 87 Europeans (CEU) and 88 Africans (YRI). After further filtering SNPs that could not match with SNPs in the 1000 genome data, or had no variation in our sample, we finally had 289,112 SNPs on the 22 autosomal chromosomes for admixture mapping.

### Admixture mapping analysis

We performed admixture mapping analysis using all the 54 EAC/BE cases as well as using just the cleaned samples comprising 28 cases.

Phases of the 54 and 28 cases were respectively inferred by Beagle 3.3.2 [18], then local African and European ancestries for each dataset were estimated with SABER [19].

Because the dataset of 54 EAC cases has a lower sample call rate than the dataset of 28 cases, we first compared the allele frequencies in the 54 individuals with those estimated from the 1000 genome data (estimated as 0.8 freqYRI+0.2 freqCEU) for the same allele at each SNP. Among the 289,112 autosomal SNPs, 908 SNPs had an allele frequency difference > 0.3. For the dataset of 54 individuals, we did two admixture mapping analyses: one using all the 289,112 SNPs, and the other excluding these 908 SNPs.

### Test of excess European or African ancestry

The incidence of esophageal adenocarcinomas in EAs is five times higher than that in AAs [13,20,21], and so any excess of European ancestry at a SNP could be detecting a region harboring susceptibility variants for EAC and BE. Conversely, such a region would not show excess African ancestry. Excess European and African ancestries were estimated and tested by the following method [22].

Assume we have a total of N individuals and L marker loci.

For the i-th individual at marker locus l, (i = 1,2, …, N; l = 1, 2, …, L), let *q*_*il*_ be the estimated European ancestry.

The excess European ancestry is estimated as ${\Delta \Pi }_{l}=\frac{1}{N}\sum_{i=1}^{N}\left(q_{il}-M_{i}\right)$, where $M_{i}=\frac{1}{L}\sum_{l=1}^{L}q_{il}$.

Then the Z score test statistic is defined as $Z_{l}=\frac{{\Delta \Pi }_{l}}{SD({\Delta \Pi }_{l})}$, where *SD*(ΔΠ_*l*_) is the common standard deviation of ΔΠ_*l*_ estimated over all the markers.

In view of the large numbers, *Z*_*l*_ follows a standard normal distribution, and the test for excess European ancestry was conducted at each locus, using a right tailed test. The test of excess African ancestry, that is, the association of esophageal adenocarcinomas being due to African ancestry, was conducted by using a left tailed test with the same statistic.

### Estimating the number of independent tests

The number of independent tests was estimated by the eigenvalue method using the local ancestry of each chromosome [23] and summed up over the 22 autosomal chromosomes. That is, for each chromosome, we calculated the eigenvalues of the correlation matrix of the local ancestry of SNPs on that chromosome, and the effective number of independent tests for that chromosome was estimated as $M=\frac{\sum_{i=1}^{n}\sqrt{\lambda_{i}}}{\sum_{i=1}^{n}\lambda_{i}}$, where *λ*_*i*_ is the *i*-th eigenvalue and *n* is the total number of eigenvalues for that chromosome.

## Results

There is no significant difference on demographic statistics between the whole samples of 54 individuals and the subset of 28 individuals (Table 1). In addition, the whole sample of 54 individuals has mean proportion (± standard error) of European ancestry 0.32±0.20, and the subset of 28 individuals has mean proportion of European ancestry 0.27±0.14; both are close to estimates of African ancestry in the literature [24,25].

**Table 1:** Demographic statistics of the admixed population of EAC/BE patients.

	All samples	Subset	P
Number of individuals	54	28	
Diagnosis (%)			1*
EAC	40 (74.1%)	21 (75.0%)	
BE	14 (25.9%)	7 (25.0%)	
Gender (%)			0.35*
Male	35 (64.8%)	16 (57.1%)	
Female	19 (35.2%)	12 (42.9%)	

* Chi square test of association between the data of 28 individuals and the remaining data of 26 (= 54–28) individuals.

### The number of independent tests

By the eigenvalue method, the number of independent tests for 54 individuals estimated using all SNPs is 288.3; after excluding the 908 SNPs it is 281.9. The number of independent tests for the 28 individuals using all SNPs is 252.2. Thus, the corresponding 5% genome-wide thresholds of association tests are respectively for the three scenarios 0.000173 (corresponding to -log_10_(P) = 3.76), 0.000177 (corresponding to -log_10_(P) = 3.75), and 0.000198 (corresponding to -log_10_(P) = 3.70).

### Excess of European ancestry

**Fig 1:**
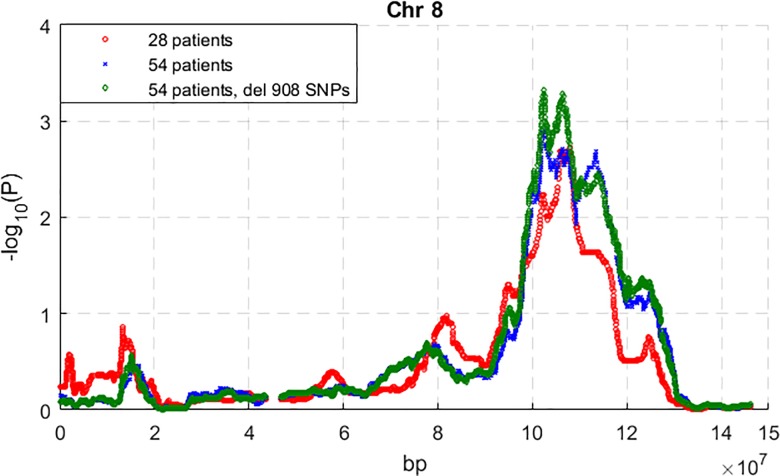
Admixture mapping test for excess European ancestry on chromosome 8 using all 54 AA patients and the subset of 28 high genotyping quality patients, respectively.

**Fig 2:**
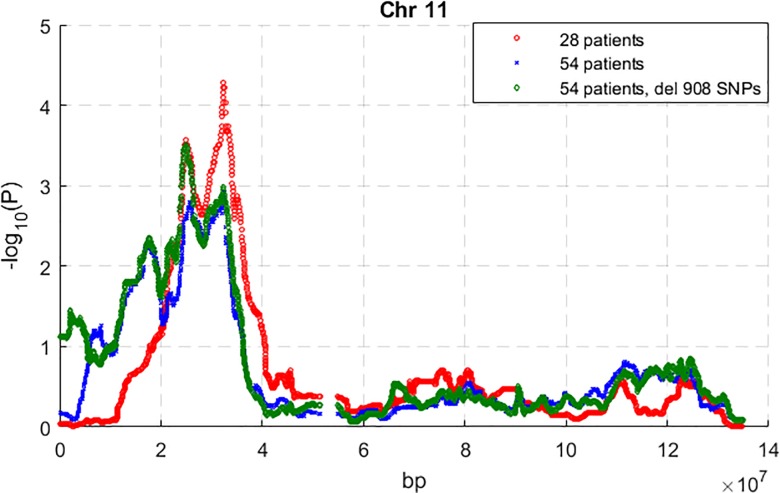
Admixture mapping test for excess European ancestry on chromosome 11 using all 54 AA patients and the subset of 28 high genotyping quality patients, respectively.

The results of genome-wide excess of European ancestry among the AA EAC/BE patients are shown in S1–S3 Figs. From all the 54 patients, removing the 908 SNPs that have allele frequency difference > 0.3 did not decrease the admixture mapping signal (S1–S3 Figs; Figs 1 and 2). The highest excess of European ancestry signals that are in common for the 54- and 28-patient datasets are on chromosomes 11 and 8 (Figs 1 and 2). From the 28 patients, the maximum association signal (-log_10_(P)) on chromosomes 8 and 11 are respectively 2.73 and 4.28. Because the genome-wide significance threshold for this dataset is 3.70, the signal on chromosome 11 reached genome-wide significance (Fig 2). From the 54 patients without the 908 SNPs, the maximum association signals (-log_10_(P)) on chromosomes 8 and 11 are respectively 3.32 and 3.51, which do not reach the genome-wide significance threshold of 3.75.

The region of excess European ancestry on chromosome 8 is from 90.4 Mb to 129 Mb, with the peak at 106.3 Mb, and the excess of European ancestry at the peak is 0.15 (S1 File). The region of excess European ancestry on chromosome 11 is from 5.6 Mb to 42.4 Mb, with the two peaks at 24.8 Mb and 32.5 Mb respectively, and the maximum mean excesses of European ancestry are 0.18 and 0.2 (S1 File).

### Excess African ancestry

No association due to excess African ancestry reached any genome-wide significance threshold for any of the three datasets; the strongest associations due to African ancestry are on chromosomes 15 and 16, but they are far from reaching genome-wide significance level: the association P-values for the three datasets are all > 0.001 (S4–S6 Figs).

It is noteworthy that, compared with the association due to European ancestry, fewer and lower association peaks due to African ancestry are found (compare S1–S3 Figs with S4–S6 Figs)

## Discussion

Admixture mapping is an effective tool for discovering genetic regions associated with disease. The method interrogates a recently admixed population such as AAs for genetic associations to diseases when prevalence differs markedly between the ancestral Caucasian and African populations [26–30]. Admixture mapping is particularly more powerful than GWAS in detecting regions that harbor rare or multiple different disease variants [31]. Admixture studies are cost effective, require less dense maps, and compared to association studies, they do not assume a disease model [31]. The identification of ancestry informative markers (AIMS) enables such approaches to be successful in diseases such as BE and EAC, whose prevalence are vastly disparate between AAs and EAs [32–38]. In this study, we performed a genome-wide case only admixture mapping association study of BE and EAC using 54 BE/EAC cases and a subset of 28 cases with high genotyping quality. We identified two chromosome regions with excess European ancestry in both datasets; one on chromosome 11p15, which reached genome-wide significance in the 28 cases, and a second one on chromosome 8q22-24. It is not surprising that the results from the two datasets are consistent, but it does suggest that the signals are not caused by genotype errors. We did not find any significant chromosome region with excess African ancestry. Because no common variants have been reported in these two regions in the genome wide association studies of BE/EAC so far, the current result indicates that these two regions with excess European ancestry likely harbor low frequency and/or rare disease associated variants that confer susceptibility to developing BE and EAC.

The fact that there are more and higher admixture mapping peaks of excess European ancestry than of excess African ancestry is consistent with the higher prevalence of BE and EAC in European Americans than in African Americans. Although not conclusive, this is certainly suggestive.

At the admixture peak on chromosome 8, we have a mean excess European ancestry of 0.15; and at the admixture peak on chromosome 11, we have a higher mean excess European ancestry of 0.2. Assuming an additive model for the genetic variants, the estimated relative risks for European alleles in the chromosome 8q and 11p regions would be 1.93 and 2.81, respectively (S1 File). However, because of the large number of regions examined, these are over-estimates due to the winner’s curse.

The admixture mapping region on 8q22-24 overlaps a linkage region that we previously identified in a linkage study [39]. It also overlaps with the 8q24 region, which is associated with multiple epithelial cancers [40–47]. This region is known to contain tissue specific enhancers that drive c-MYC expression in colon cancers [42,48,49]. Moreover, there are other known cancer susceptibility genes located in the two admixture mapping regions we identified. Metadherin (MTDH), also known as Astrocyte elevated gene-1 (AEG-1), locates 7 Mb from the peak of the chromosome 8 region. It plays an important role in carcinogenesis [50], and overexpression of MTDH/AEG-1 can significantly enhance cell proliferation and anchorage-independent growth ability in a variety of cancers [51]. The Wilms’ tumor gene WT1 locates at the rightmost (the highest) peak on chromosome 11; it is a tumor-suppressor gene [52] that encodes a zinc finger transcription factor regulating transcription of growth factors such as PDGF-A [53], growth factor receptor (IGF-IR) [54] and other genes (RAR-α, c-myc and bcl-2) [55,56]. Myogenic differentiation 1 MYOD1 is 7 Mb to the left of the peak on chromosome 11, and was reported to have frequent hypermethylation in intestinal metaplasia tissue (Barrett’s esophagus) [57]. The oncogene MYC [58] is close to our chromosome 8 admixture mapping region and, because the location of genetic variants for BE/EAC in our study could be inaccurately estimated due to the small sample size, this is another candidate gene for further fine mapping study. The admixture mapping regions on the two chromosomes are large, with more than 200 genes in the chromosome 8 region and more than 300 genes in the chromosome 11 region, making it is hard to locate the susceptibility genes in this kind of study. Moreover, the susceptibility variants could be miRNAs, other non-coding RNAs, or regulatory elements in the regions. The identification of the susceptibility genes and variants will require a large-scale accrual of cases at multiple centers since AA EAC cases are rare.

The results of this study indicate the power of the admixture approach for racially disparate diseases such as BE and EAC. The case only admixture mapping study is a more efficient design for a rare disease such as EAC than case-control design [26] and, because it is based on comparing local ancestry with average local or global ancestry [27, 59], it is robust against any population stratification difference between cases and controls. The rarity of BE and EAC limited our available sample size. Furthermore, the quality of the fragmented DNA extracted from archived FFPE blocks resulted in poor quality SNP calls for nearly half our available samples. Due to the limitations of the small sample size and the DNA quality, our admixture results could miss other regions and the case only admixture mapping could not accurately locate association variants. Despite these limitations, the admixture analysis was able to identify two regions of excess European ancestry that appear to be associated with BE and EAC. It is especially noteworthy that poor sample quality, which would seriously affect association results, has less of a detrimental effect on admixture mapping (Figs 1 and 2); this can be explained by the fact that in admixture mapping larger regions are studied, and within such regions positive and negative errors tend to cancel out. This, of course, comes at the expense of a less precise location of any causal variant.

In conclusion, our admixture mapping association study of BE and EAC identified chromosome 8q22-24 and chromosome 11p15 with excess European ancestry. This is the first admixture analysis to suggest a genetic basis for the racial disparity in the prevalence of BE and EAC. One possible mechanism to explain racial disparity in BE and EAC is that the normal squamous mucosa of esophagus in AA is less susceptible to refluxate damage, and subsequent development of metaplastic BE, than that of EA. This might be secondary to endogenous protection from more active detoxifying enzymes, more extensive mucin production, or the increased expression of protective gene products in AA. A second possible mechanism is that Barrett’s epithelium is more likely to replace damaged squamous epithelium in EA than AA. Thus, admixed AA cases with BE may carry European alleles that more readily replace damaged squamous epithelium with metaplastic BE. Further sequencing of the two regions with a larger sample size will be conducted to locate genetic variants for esophageal adenocarcinoma and Barrett’s esophagus and identify the mechanisms that explain resistance to the development of BE in individuals of African ancestry.

## Supporting information

S1 Fig The genome-wide admixture mapping result for excess European ancestry signals from 54 individuals using all SNPs. (TIF)

S2 Fig The genome-wide admixture mapping result for excess European ancestry signals from 54 individuals using SNPs without the 908 SNPs having allele frequencies different from the 1000 genomes. (TIF)

S3 Fig The genome-wide admixture mapping result for excess European ancestry signals from the 28 individuals having high genotyping quality. (TIF)

S4 Fig The genome-wide admixture mapping result for excess African ancestry signals from 54 individuals using all SNPs. (TIF)

S5 Fig The genome-wide admixture mapping result for excess African ancestry signals from 54 individuals excluding the 908 SNPs having different allele frequency from the 1000 genomes. (TIF)

S6 Fig The genome-wide admixture mapping result for excess African ancestry signals from the 28 individuals having high genotyping quality. (TIF)

S1 File Estimating the association effect size of variants in the two admixture mapping regions. (DOCX)

## References

[1] American Cancer Society. Cancer Facts & Figures 2017. Atlanta: American Cancer Society; 2017

[2] Holmes R.S, Vaughan TL. Epidemiology and pathogenesis of esophageal cancer. Semin Radiat Oncol. 2007;17: 2–9. doi: 10.1016/j.semradonc.2006.09.003 17185192 10.1016/j.semradonc.2006.09.003

[3] Pohl H, Welch HG. The role of overdiagnosis and reclassification in the marked increase of esophageal adenocarcinoma incidence. J Natl Cancer Inst. 2005;97: 142–146. doi: 10.1093/jnci/dji024 15657344 10.1093/jnci/dji024

[4] Brown LM, Devesa SS, Chow WH. Incidence of adenocarcinoma of the esophagus among white Americans by sex, stage, and age. J Natl Cancer Inst. 2008;100:1184–1187. doi: 10.1093/jnci/djn211 18695138 10.1093/jnci/djn211 PMC2518165

[5] Chak A, Ochs-Balcom H, Falk G, Grady WM, Kinnard M, Willis JE, et al Familiality in Barrett's esophagus, adenocarcinoma of the esophagus, and adenocarcinoma of the gastroesophageal junction. Cancer Epidemiol Biomarkers Prev. 2006;15: 1668–1673. doi: 10.1158/1055-9965.EPI-06-0293 16985029 10.1158/1055-9965.EPI-06-0293

[6] Altorki NK, Skinner DB. Adenocarcinoma in Barrett's esophagus. Semin Surg Oncol. 1990;6: 274–278. 2237086 10.1002/ssu.2980060509

[7] Cameron AJ, Lomboy CT. Barrett's esophagus: age, prevalence, and extent of columnar epithelium. Gastroenterology. 1992;103: 1241–1245. 1397881 10.1016/0016-5085(92)91510-b

[8] Falk GW. Barrett's esophagus. Gastroenterology. 2002;122: 1569–1591. 12016424 10.1053/gast.2002.33427

[9] Anderson LA, Watson RG, Murphy SJ, Johnston BT, Comber H, Mc Guigan J, et al Risk factors for Barrett's oesophagus and oesophageal adenocarcinoma: results from the FINBAR study. World J Gastroenterol. 2007;13: 1585–1594. doi: 10.3748/wjg.v13.i10.1585 17461453 10.3748/wjg.v13.i10.1585 PMC4146903

[10] DeVault KR. Epidemiology and significance of Barrett's esophagus. Dig Dis. 2000;18: 195–202. 11356990 10.1159/000051399

[11] Edelstein ZR, Bronner MP, Rosen SN, Vaughan TL. Risk factors for Barrett's esophagus among patients with gastroesophageal reflux disease: a community clinic-based case-control study. Am J Gastroenterol. 2009;104: 834–842. doi: 10.1038/ajg.2009.137 19319131 10.1038/ajg.2009.137 PMC2714477

[12] Falk GW. Risk factors for esophageal cancer development. Surg Oncol Clin N Am. 2009;18: 469–485. doi: 10.1016/j.soc.2009.03.005 19500737 10.1016/j.soc.2009.03.005

[13] Cook MB, Chow WH, Devesa SS. Oesophageal cancer incidence in the United States by race, sex, and histologic type, 1977–2005. Br J Cancer. 2009;101: 855–859. doi: 10.1038/sj.bjc.6605246 19672254 10.1038/sj.bjc.6605246 PMC2736840

[14] Sharma P, Wani S, Romero Y, Johnson D, Hamilton F. Racial and geographic issues in gastroesophageal reflux disease. Am J Gastroenterol. 2008;103: 2669–2680. doi: 10.1111/j.1572-0241.2008.02089.x 19032462 10.1111/j.1572-0241.2008.02089.x

[15] Ogden CL, Carroll MD, Kit BK, Flegal KM. Prevalence of obesity among adults: United States, 2011–2012. NCHS Data Brief. 2013: 1–8. 24152742

[16] An R. Prevalence and Trends of Adult Obesity in the US, 1999–2012. ISRN Obes. 2014;2014: 185132. 10.1155/2014/185132 PMC3913362 25002986

[17] 1000 Genomes Project Consortium, Auton A, Brooks LD, Durbin RM, Garrison EP, Kang HM, Korbel JO, et al A global reference for human genetic variation. Nature. 2015;526: 68–74. doi: 10.1038/nature15393 26432245 10.1038/nature15393 PMC4750478

[18] Browning SR, Browning BL. Rapid and accurate haplotype phasing and missing data inference for whole genome association studies using localized haplotype clustering. Am J Hum Genet. 2007;81: 1084–1097. doi: 10.1086/521987 17924348 10.1086/521987 PMC2265661

[19] Johnson NA, Coram MA, Shriver MD, Romieu I, Barsh GS, London SJ, et al Ancestral components of admixed genomes in a Mexican cohort. PLoS Genet. 2011;7: e1002410 doi: 10.1371/journal.pgen.1002410 22194699 10.1371/journal.pgen.1002410 PMC3240599

[20] Lepage C, Drouillard A, Jouve JL, Faivre J. Epidemiology and risk factors for oesophageal adenocarcinoma. Dig Liver Dis. 2013;45: 625–629. doi: 10.1016/j.dld.2012.12.020 23453359 10.1016/j.dld.2012.12.020

[21] Baquet CR, Commiskey P, Mack K, Meltzer S, Mishra SI. Esophageal cancer epidemiology in blacks and whites: racial and gender disparities in incidence, mortality, survival rates and histology. J Natl Med Assoc. 2005;97: 1471–1478. 16334494 PMC2594901

[22] Zhu X, Luke A, Cooper RS, Quertermous T, Hanis C, Mosley T, et al Admixture mapping for hypertension loci with genome-scan markers. Nat Genet. 2005;37: 177–1781. doi: 10.1038/ng1510 15665825 10.1038/ng1510

[23] Galwey NW. A new measure of the effective number of tests, a practical tool for comparing families of non-independent significance tests. Genet Epidemiol. 2009;33: 559–568. doi: 10.1002/gepi.20408 19217024 10.1002/gepi.20408

[24] Bryc K, Durand EY, Macpherson JM, Reich D, Mountain JL. The genetic ancestry of African Americans, Latinos, and European Americans across the United States. Am J Hum Genet. 2015;96: 37–53. doi: 10.1016/j.ajhg.2014.11.010 25529636 10.1016/j.ajhg.2014.11.010 PMC4289685

[25] Zakharia F, Basu A, Absher D, Assimes TL, Go AS, Hlatky MA, et al Characterizing the admixed African ancestry of African Americans. Genome Biol. 2009;10: R141 doi: 10.1186/gb-2009-10-12-r141 20025784 10.1186/gb-2009-10-12-r141 PMC2812948

[26] Hoggart CJ, Shriver MD, Kittles RA, Clayton DG, McKeigue PM. Design and analysis of admixture mapping studies. Am J Hum Genet. 2004;74: 965–978. doi: 10.1086/420855 15088268 10.1086/420855 PMC1181989

[27] Shriner D. Overview of admixture mapping. Curr Protoc Hum Genet. 2013;Chapter 1: Unit 1.23. 10.1002/0471142905.hg0123s76 PMC3556814 23315925

[28] Chakraborty R, Weiss KM. Admixture as a tool for finding linked genes and detecting that difference from allelic association between loci. Proc Natl Acad Sci U S A. 1988;85: 9119–9123. 3194414 10.1073/pnas.85.23.9119 PMC282675

[29] Briscoe D, Stephens JC, O'Brien SJ. Linkage disequilibrium in admixed populations: applications in gene mapping. J Hered. 1994;85: 59–63. 8120361

[30] Zhu X, Tang H, Risch N. Admixture mapping and the role of population structure for localizing disease genes. Adv Genet. 2008;60: 547–569. doi: 10.1016/S0065-2660(07)00419-1 18358332 10.1016/S0065-2660(07)00419-1

[31] Montana G, Pritchard JK. Statistical tests for admixture mapping with case-control and cases-only data. Am J Hum Genet. 2004;75: 771–789. doi: 10.1086/425281 15386213 10.1086/425281 PMC1182107

[32] Freedman ML, Haiman CA, Patterson N, McDonald GJ, Tandon A, Waliszewska A, et al Admixture mapping identifies 8q24 as a prostate cancer risk locus in African-American men. Proc Natl Acad Sci U S A. 2006;103: 14068–14073. doi: 10.1073/pnas.0605832103 16945910 10.1073/pnas.0605832103 PMC1599913

[33] Reich D, Patterson N, De Jager PL, McDonald GJ, Waliszewska A, Tandon A, et al A whole-genome admixture scan finds a candidate locus for multiple sclerosis susceptibility. Nat Genet. 2005;37: 1113–1118. doi: 10.1038/ng1646 16186815 10.1038/ng1646

[34] Kopp JB, Smith MW, Nelson GW, Johnson RC, Freedman BI, Bowden DW, et al MYH9 is a major-effect risk gene for focal segmental glomerulosclerosis. Nat Genet. 2008;40: 1175–1184. doi: 10.1038/ng.226 18794856 10.1038/ng.226 PMC2827354

[35] Elbein SC, Das SK, Hallman DM, Hanis CL, Hasstedt SJ. Genome-wide linkage and admixture mapping of type 2 diabetes in African American families from the American Diabetes Association GENNID (Genetics of NIDDM) Study Cohort. Diabetes. 2009;58: 268–274. doi: 10.2337/db08-0931 18840782 10.2337/db08-0931 PMC2606884

[36] Reich D, Patterson N, Ramesh V, De Jager PL, McDonald GJ, Tandon A, et al Admixture mapping of an allele affecting interleukin 6 soluble receptor and interleukin 6 levels. Am J Hum Genet. 2007;80: 716–726. doi: 10.1086/513206 17357077 10.1086/513206 PMC1852718

[37] Shetty PB, Tang H, Feng T, Tayo B, Morrison AC, Kardia SL, et al Variants for HDL-C, LDL-C, and triglycerides identified from admixture mapping and fine-mapping analysis in African American families. Circ Cardiovasc Genet. 2015;8: 106–113. doi: 10.1161/CIRCGENETICS.114.000481 25552592 10.1161/CIRCGENETICS.114.000481 PMC4378661

[38] Shetty PB, Tang H, Tayo BO, Morrison AC, Hanis CL, Rao DC, et al Variants in CXADR and F2RL1 are associated with blood pressure and obesity in African-Americans in regions identified through admixture mapping. J Hypertens. 2012;30: 1970–1976. doi: 10.1097/HJH.0b013e3283578c80 22914544 10.1097/HJH.0b013e3283578c80 PMC3575678

[39] Sun X, Elston R, Falk GW, Grady WM, Faulx A, Mittal SK, et al Linkage and related analyses of Barrett's esophagus and its associated adenocarcinomas. Mol Genet Genomic Med. 2016 4:407–419. doi: 10.1002/mgg3.211 27468417 10.1002/mgg3.211 PMC4947860

[40] Han Y, Rand KA, Hazelett DJ, Ingles SA, Kittles RA, Strom SS, et al Prostate Cancer Susceptibility in Men of African Ancestry at 8q24. J Natl Cancer Inst. 2016;108. 10.1093/jnci/djv431 PMC4948565 26823525

[41] Raeder MB, Birkeland E, Trovik J, Krakstad C, Shehata S, Schumacher S, et al Integrated genomic analysis of the 8q24 amplification in endometrial cancers identifies ATAD2 as essential to MYC-dependent cancers. PLoS One. 2013;8: e54873 doi: 10.1371/journal.pone.0054873 23393560 10.1371/journal.pone.0054873 PMC3564856

[42] Brisbin AG, Asmann YW, Song H, Tsai YY, Aakre JA, Yang P, et al Meta-analysis of 8q24 for seven cancers reveals a locus between NOV and ENPP2 associated with cancer development. BMC Med Genet. 2011;12: 156 doi: 10.1186/1471-2350-12-156 22142333 10.1186/1471-2350-12-156 PMC3267702

[43] Yeager M, Chatterjee N, Ciampa J, Jacobs KB, Gonzalez-Bosquet J, Hayes RB, et al Identification of a new prostate cancer susceptibility locus on chromosome 8q24. Nat Genet. 2009;41: 1055–1057. doi: 10.1038/ng.444 19767755 10.1038/ng.444 PMC3430510

[44] Pomerantz MM, Beckwith CA, Regan MM, Wyman SK, Petrovics G, Chen Y, et al Evaluation of the 8q24 prostate cancer risk locus and MYC expression. Cancer Res. 2009;69: 5568–5574. doi: 10.1158/0008-5472.CAN-09-0387 19549893 10.1158/0008-5472.CAN-09-0387 PMC2884104

[45] Penney KL, Salinas CA, Pomerantz M, Schumacher FR, Beckwith CA, Lee GS, et al Evaluation of 8q24 and 17q risk loci and prostate cancer mortality. Clin Cancer Res. 2009;15: 3223–3230. doi: 10.1158/1078-0432.CCR-08-2733 19366828 10.1158/1078-0432.CCR-08-2733 PMC2878092

[46] Schumacher FR, Feigelson HS, Cox DG, Haiman CA, Albanes D, Buring J, et al A common 8q24 variant in prostate and breast cancer from a large nested case-control study. Cancer Res. 2007;67: 2951–2956. doi: 10.1158/0008-5472.CAN-06-3591 17409400 10.1158/0008-5472.CAN-06-3591

[47] Yeager M, Orr N, Hayes RB, Jacobs KB, Kraft P, Wacholder S, et al Genome-wide association study of prostate cancer identifies a second risk locus at 8q24. Nat Genet. 2007;39: 645–649. doi: 10.1038/ng2022 17401363 10.1038/ng2022

[48] Ahmadiyeh N, Pomerantz MM, Grisanzio C, Herman P, Jia L, Almendro V, et al 8q24 prostate, breast, and colon cancer risk loci show tissue-specific long-range interaction with MYC. Proc Natl Acad Sci U S A. 2010;107: 9742–9746. doi: 10.1073/pnas.0910668107 20453196 10.1073/pnas.0910668107 PMC2906844

[49] Tuupanen S, Turunen M, Lehtonen R, Hallikas O, Vanharanta S, Kivioja T, et al The common colorectal cancer predisposition SNP rs6983267 at chromosome 8q24 confers potential to enhanced Wnt signaling. Nat Genet. 2009;41: 885–890. doi: 10.1038/ng.406 19561604 10.1038/ng.406

[50] Shi X, Wang X. The role of MTDH/AEG-1 in the progression of cancer. Int J Clin Exp Med. 2015;8: 4795–4807. 26131054 PMC4484038

[51] Yu C, Chen K, Zheng H, Guo X, Jia W, Li M, et al Overexpression of astrocyte elevated gene-1 (AEG-1) is associated with esophageal squamous cell carcinoma (ESCC) progression and pathogenesis. Carcinogenesis. 2009;30: 894–901. doi: 10.1093/carcin/bgp064 19304953 10.1093/carcin/bgp064

[52] Oji Y, Yano M, Nakano Y, Abeno S, Nakatsuka S, Ikeba A, et al Overexpression of the Wilms' tumor gene WT1 in esophageal cancer. Anticancer Res. 2004;24: 3103–3108. 15510596

[53] Gashler AL, Bonthron DT, Madden SL, Rauscher FJ 3rd, Collins T, Sukhatme VP. Human platelet derived growth factor A chain is transcriptionally repressed by the Wilms’ tumor suuppressor WT1. Proc Natl Acad Sci U S A. 1992;89: 10984–10988. 1332065 10.1073/pnas.89.22.10984 PMC50467

[54] Werner H, Re GG, Drummond IA, Sukhatme VP, Rauscher FJ 3rd, Sens DA, et al Increased expression of the insulin-like growth factor I receptor gene, IGFIR, in Wilms’ tumor is correlated with modulation of IGFIR promoter activity by the WT1 Wilms’ tumor gene product. Proc Natl Acad Sci U S A. 1993;90: 5828–5832. 8390684 10.1073/pnas.90.12.5828 PMC46816

[55] Goodyer P, Dehbi M, Torban E, Bruening W, Pelletier J. Repression of the retinoic acid receptor-alpha gene by the Wilms' tumor suppressor gene product, WT1. Oncogene. 1995;10: 1125–1129. 7700638

[56] Hewitt SM, Hamada S, McDonnell TJ, Rauscher FJ 3rd, Saunders GF. Regulation of the proto-oncogenes bcl-2 and c-myc by the Wilms' tumor suppressor gene WT1. Cancer Res. 1995;55: 5386–5389. 7585606

[57] Eads CA, Lord RV, Wickramasinghe K, Long TI, Kurumboor SK, Bernstein L, et al Epigenetic patterns in the progression of esophageal adenocarcinoma. Cancer Res. 2001;61: 3410–3418. 11309301

[58] Sotelo J, Esposito D, Duhagon MA, Banfield K, Mehalko J, Liao H, et al Long-range enhancers on 8q24 regulate c-Myc. Proc Natl Acad Sci U S A. 2010;107: 3001–3005. doi: 10.1073/pnas.0906067107 20133699 10.1073/pnas.0906067107 PMC2840341

[59] Shriner D, Adeyemo A, Ramos E, Chen G, Rotimi CN. Mapping of disease-associated variants in admixed populations. Genome Biol. 2011;12: 223 doi: 10.1186/gb-2011-12-5-223 21635713 10.1186/gb-2011-12-5-223 PMC3219963

