# Supplementary figures and images for: Genomic regions associated with susceptibility to Barrett’s esophagus and esophageal adenocarcinoma in African Americans: The cross BETRNet admixture study
Source: PLoS One. 2017 Oct 26;12(10):e0184962. doi: 10.1371/journal.pone.0184962 (PMC5657624; doi:10.1371/journal.pone.0184962)

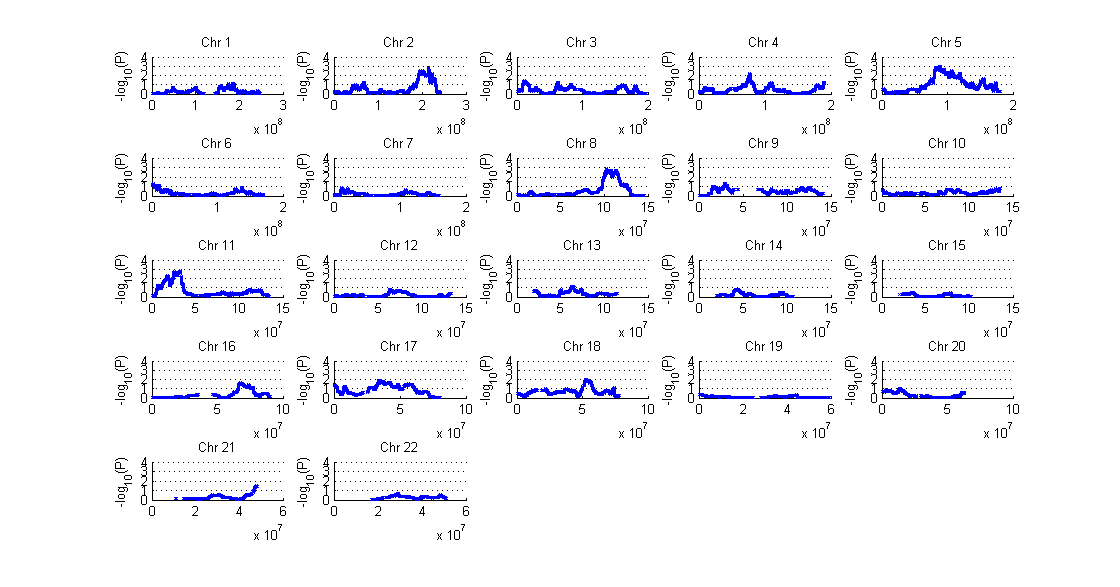

Supplement: S1 Fig — (TIF) [file pone.0184962.s001.tif]

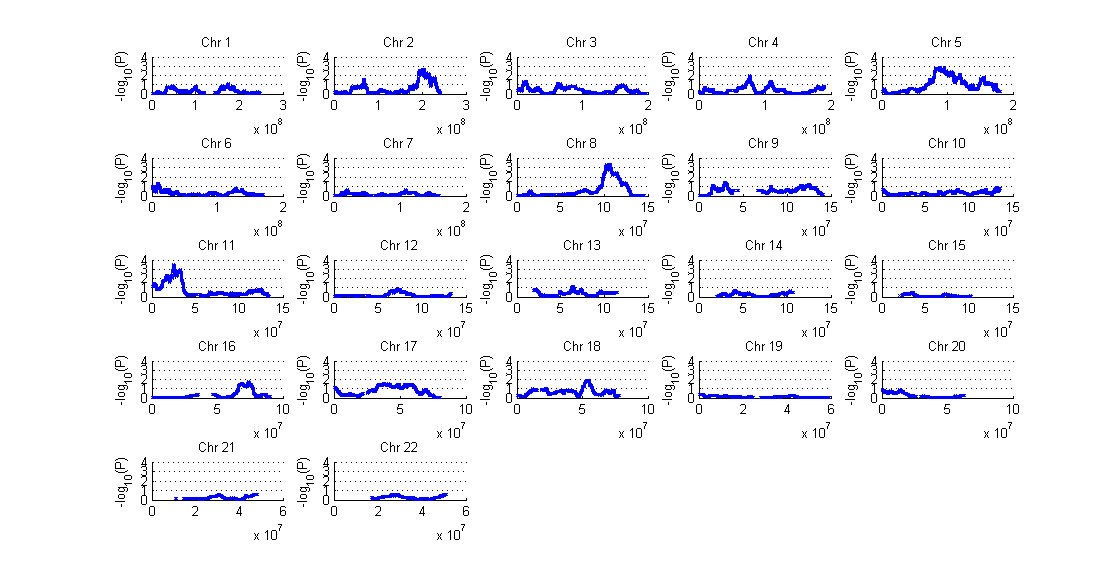

Supplement: S2 Fig — (TIF) [file pone.0184962.s002.tif]

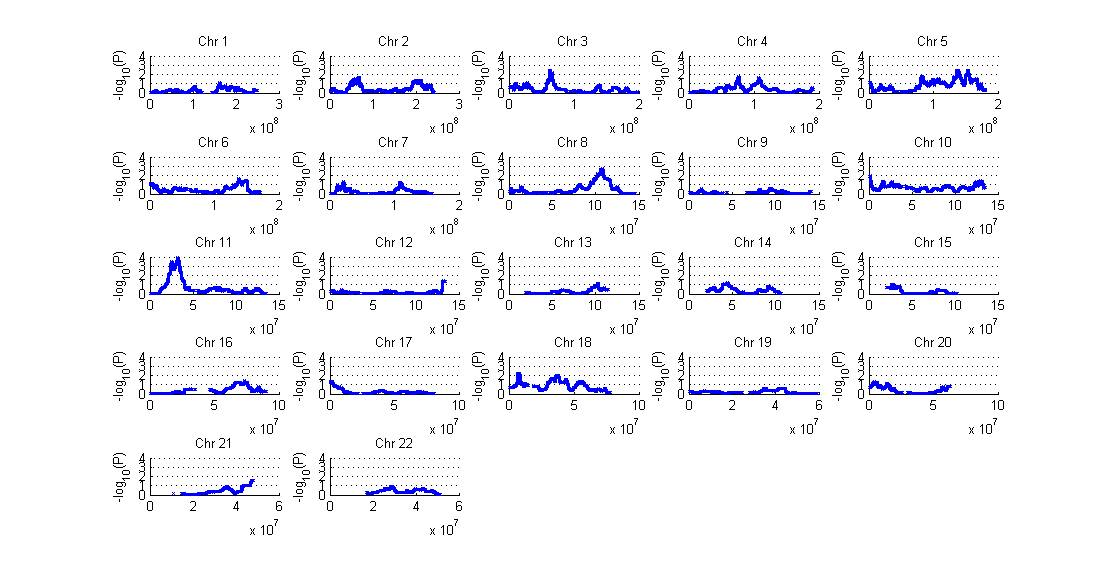

Supplement: S3 Fig — (TIF) [file pone.0184962.s003.tif]

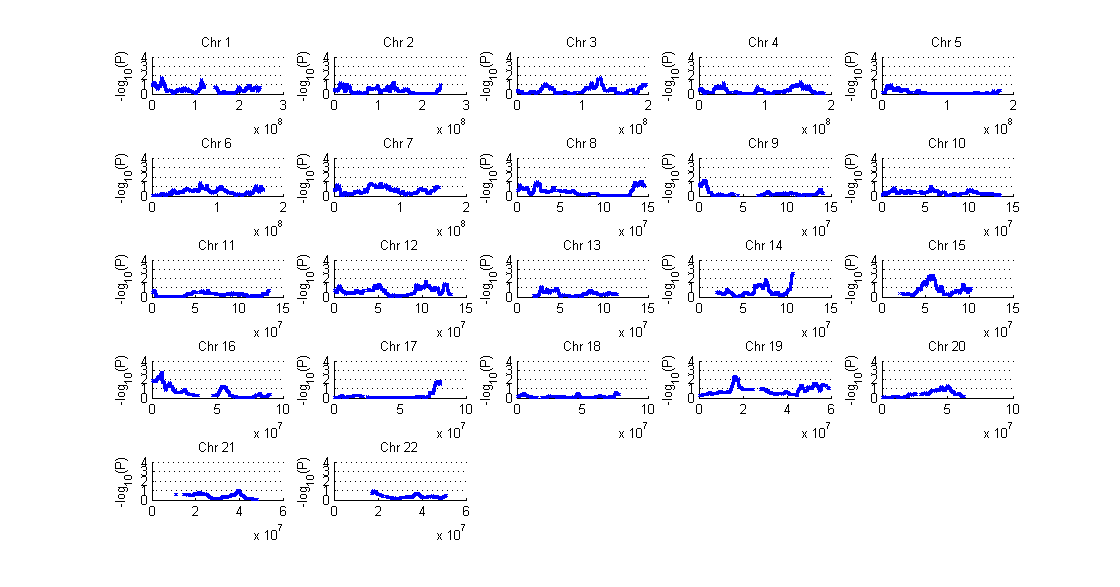

Supplement: S4 Fig — (TIF) [file pone.0184962.s004.tif]

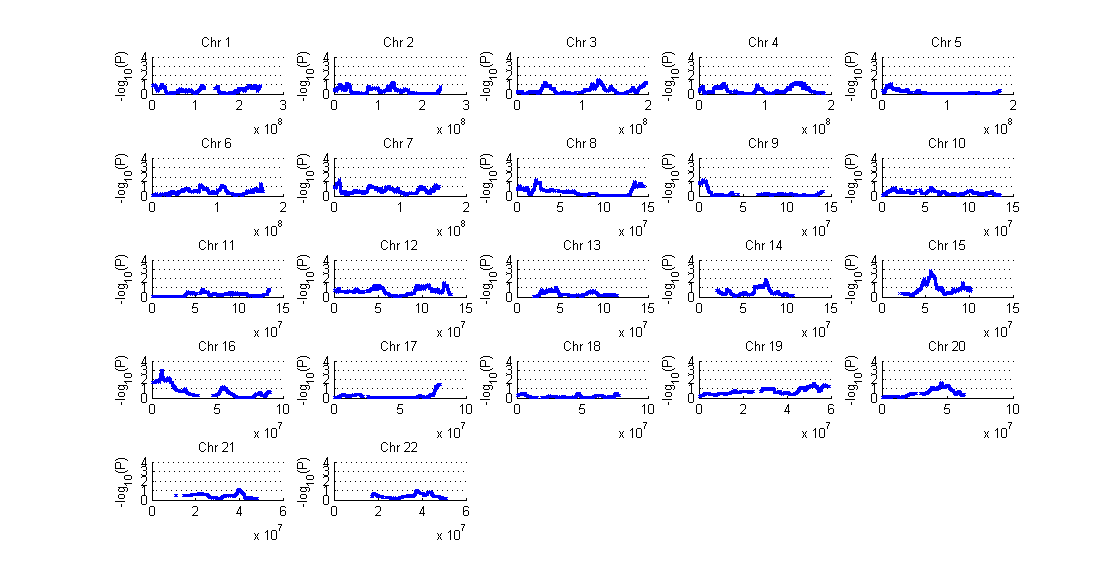

Supplement: S5 Fig — (TIF) [file pone.0184962.s005.tif]

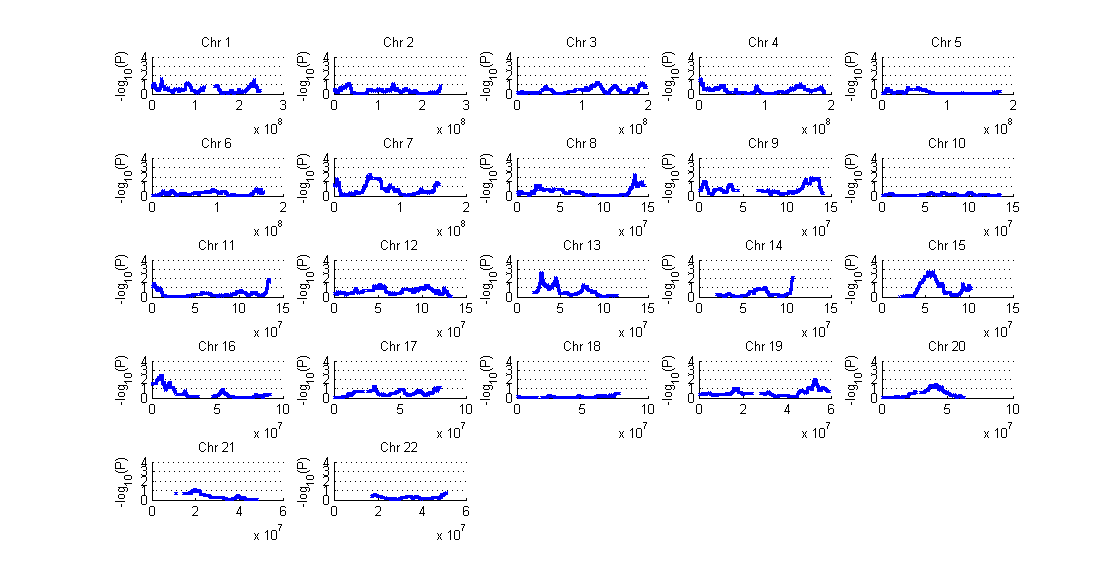

Supplement: S6 Fig — (TIF) [file pone.0184962.s006.tif]
