# Supplementary material for: Genomic regions associated with susceptibility to Barrett’s esophagus and esophageal adenocarcinoma in African Americans: The cross BETRNet admixture study
Source: PLoS One. 2017 Oct 26;12(10):e0184962. doi: 10.1371/journal.pone.0184962 (PMC5657624; doi:10.1371/journal.pone.0184962)
Supplement: S1 File — (DOCX) [file pone.0184962.s007.docx]

**Estimating the association effect size of variants in the two admixture mapping regions**

According to the admixture mapping result of the 28 BE and EAC cases, at the admixture peak on chromosome 8 we have an average excess European ancestry of 0.148, and at the admixture peak of chromosome 11 we have a higher average excess European ancestry of 0.198. With these results, we show how the relative risk of the European ancestry allele can be approximately estimated at the admixture peaks (ignoring the effect of the winner’s curse).

In our data, the mean European ancestry in the population is P(E) = 0.27, where E denotes European ancestry.

**1. Inferring the relative risk on chromosome 11**

The mean excess of European ancestry at the peak on chromosome 11 is P(excess E|case) = 0.198, so the mean European ancestry in cases is

P(E|case) = P(excess E|case) + P(E) ≈ 0.20+0.27 = 0.47.

Now P(E|case) = P(E, case)/P(case), (1)

where P(E,case) = P(EE, case) + 0.5*P(EA, case) = P(case|EE)P(EE) + 0.5P(case|EA)P(EA), with EE, EA, and AA denoting the EE, EA, and AA genotypes, respectively;

and P(case) = P(case|EE)P(EE) + P(case|EA)P(EA) + P(case|AA)P(AA).

So (1) becomes

P(E|case) = [P(case|EE)P(EE) + 0.5P(case|EA)P(EA)]/[P(case|EE)P(EE) + P(case|EA)P(EA) + P(case|AA)P(AA)] (2)

Dividing the numerator and the denominator of (2) by P(case|AA), and defining the relative risks r_EE_ = P(case|EE)/ P(case|AA) and r_EA_ = P(case|EA)/ P(case|AA), we have

P(E|case) = [r_EE_P(EE) + r_EA_(0.5* P(EA))]/[ r_EE_P(EE) + r_EA_P(EA) + 1*P(AA)] = 0.47 (3)

- 1. **Assuming additive risk**

Assuming r_EE_ = 2r_EA_, then from (3) we have

2r_EA_ P(EE) + r_EA_(0.5* P(EA)) = 0.47*[ 2r_EA_ P(EE) + r_EA_P(EA) + 1*P(AA)],

so r_EA_ = 0.47 P(AA)/[(2-2*0.47)P(EE) + (0.5-0.47)P(EA)].

By assuming HWE at the risk locus, P(EE) = P(E)^2^, P(EA) = 2P(E)(1- P(E)), and P(AA) = (1-P(E))^2^, so

r_EA_ = 0.47(1-P(E))^2^/[(2-2*0.47) P(E)^2^ + (0.5-0.47) 2P(E)(1- P(E))] = 0.47*(1-0.27)^2^/(2*0.53*0.27^2^ + 0.03*2*0.27*0.73)

≈ 2.81

r_EE_ ≈ 5.62

- 1. **Assuming multiplicative risk**

Assuming r_EE_ = r_EA_^2^, then from (3) we have

r_EA_^2^ P(EE) + r_EA_(0.5* P(EA)) = 0.47*[ r_EA_^2^ P(EE) + r_EA_P(EA) + 1*P(AA)]

P(EE) (1-0.47) r_EA_^2^ + (0.5-0.47) P(EA) r_EA_ – 0.47 P(AA) = 0

0.27^2^ (1– 0.47) r_EA_^2^ + 0.03*2*0.27*0.73 r_EA_ – 0.47*0.73^2^ = 0

0.039 r_EA_^2^ + 0.012 r_EA_ – 0.240 = 0

r_EA_ ≈ 2.40

r_EE_ ≈ 5.75

So if the risk locus is additive on chromosome 11, the relative risk of the European allele will be 2.81; and assuming a multiplicative risk locus on chromosome 11, the relative risk of the European allele will be 2.40.

**2. Inferring the relative risk on chromosome 8**

On chromosome 8, P(excess E|case) = 0.148; so P(E|case) = P(excess E|case) + P(E) ≈ 0.15 +0.27 = 0.42.

**2.1. Assuming additive risk**

Assuming r_EE_ = 2r_EA_, then from (3) we have

2r_EA_ P(EE) + r_EA_(0.5* P(EA)) = 0.42*[ 2r_EA_ P(EE) + r_EA_P(EA) + 1*P(AA)],

so r_EA_ = 0.42 P(AA)/[(2-2*0.42)P(EE) + (0.5-0.42)P(EA)].

By assuming HWE at the risk locus, P(EE) = P(E)^2^, P(EA) = 2P(E)(1- P(E)), and P(AA) = (1-P(E))^2^,

r_EA_ = 0.42(1-P(E))^2^/[(2-2*0.42) P(E)^2^ + (0.5-0.42) 2P(E)(1- P(E))] = 0.42*(1-0.27)^2^/(2*0.58*0.27^2^ + 0.08*2*0.27*0.73)

≈ 1.93

r_EE_ ≈ 3.86

**2.2. Assuming multiplicative risk**

Assuming r_EE_ = r_EA_^2^, then from (3) we have

r_EA_^2^ P(EE) + r_EA_(0.5* P(EA)) = 0.42*[ r_EA_^2^ P(EE) + r_EA_P(EA) + 1*P(AA)]

P(EE) (1-0.42) r_EA_^2^ + (0.5-0.42) P(EA) r_EA_ – 0.42 P(AA) = 0

0.27^2^ (1– 0.42) r_EA_^2^ + 0.08*2*0.27*0.73 r_EA_ – 0.42*0.73^2^ = 0

0.042 r_EA_^2^ + 0.032 r_EA_ – 0.224 = 0

r_EA_ ≈ 1.96

r_EE_ ≈ 3.92

So if the risk locus is additive on chromosome 8, the relative risk of the European allele will be 1.93; and assuming a multiplicative risk locus on chromosome 8, the relative risk of the European allele will be 1.96.
